# Weak Hydrogen Bond with Iodide Modulating Crystallization of Methylammonium Lead Iodide for High-Performance Perovskite Solar Cells

**DOI:** 10.3390/mi17010015

**Published:** 2025-12-24

**Authors:** Ning Kang, Lu Li, Zhe Wan, Liping Yang, Zhen Liang, Li Chen, Peng Li, Yongrong Sun, Zuyong Wang, Chenglong Wang

**Affiliations:** 1 National Engineering Research Center for Technology and Equipment of Environmental Deposition, Solar Thermal Industry Research Institute of Gansu Province, Lanzhou Jiaotong University, Lanzhou 730070, China; lilu200211@163.com (L.L.); zwan0122sci@gmail.com (Z.W.); yangli285731@163.com (L.Y.); lzjtdx0427@163.com (Z.L.); lichzhen@163.com (L.C.); lipeng290312@163.com (P.L.); 2 Guang Dong Engineering Technology Research Center of Biomaterials, Institute of Biological and Medical Engineering, Guangdong Academy of Sciences, Guangzhou 510316, China; sunyongronghlj@163.com; 3 College of Materials Science and Engineering, Hunan University, Changsha 410082, China; wangzy@hnu.edu.cn

**Keywords:** perovskite solar cells, glycerol, hydrogen bond involving iodide, crystallization rate, power conversion efficiency

## Abstract

The weak hydrogen bond with methylammonium iodide (MAI) dominates the formation of methylammonium lead iodide (MAPbI_3_) during its nucleation and growth process. Herein, a weak hydrogen bond involving iodide is designed between the MAI and glycerol molecule in mixed solvents containing N, N-dimethylformamide (DMF) and dimethyl sulfoxide (DMSO) to delay the growth of MAPbI_3_ film. Incorporation of glycerol into the perovskite film indicates a larger grain size and suppressed nonradiative recombination of carriers in the film. Finally, the glycerol-doped perovskite solar cells (PSCs) achieve a champion power conversion efficiency (PCE) of up to 16.84%, with excellent stability to retain 92.05% of their initial PCE after 30 days of storage. The above results unveil a deep understanding of weak hydrogen bonds in high-performance perovskite photovoltaics.

## 1. Introduction

Organic–inorganic hybrid metal halide perovskite solar cells (PSCs) have become promising photovoltaics by virtue of the low cost of their fabrication and their high power conversion efficiency (PCE), which is up to 27.2%, owing to the exceptional characteristics of perovskite materials, including a high molar extinction coefficient, long charge-carrier diffusion distance, appropriate direct band gap and tolerable defect density [1,2,3,4,5]. Anti-solvent and two-step spin-coating engineering are often employed to deposit halide perovskite films. However, surface or inner voids and defects of the MAPbI_3_ film are often inevitable, owing to the distinctive crystallization rate between organic cations (such as methylammonium (MA^+^)) and inorganic components (such as PbI_2_) during the growth process of the film [6,7]. Therefore, balancing this discrepancy of crystallization rate can compensate for the defects of the MAPbI_3_ film for high-performance PSCs.

Additive engineering has become a pivotal strategy to modulate the crystallization rate of MAPbI_3_ film. For instance, metal salts including potassium iodide (KI), potassium chloride (KCl) and lanthanide-based oleate salts have been successfully applied to modulate the growth process of perovskite film. Metal ions from these salts can inhibit the halogen ion migration and reduce the occurrence of defects at the interface by coordinating with the halogen on the surface of perovskite crystals [8,9,10,11,12]. In addition, small molecular additives (such as fluoropyridinic acid, para-tert-butylbenzoic acid (tB-COOH) and ethylamine) with various functional groups, including -NH_2_ or -COOH, exhibit a comparable effect to metal salts by forming hydrogen bonds between Pb^2+^ and functional groups to decrease the surface crystal defects of perovskite films [13,14,15,16,17]. Therefore, the hydrogen bond formed between the additive molecules and precursors (PbI_2_ or MAI for MAPbI_3_-based PSCs) is conducive to the relatively low crystallization rate of perovskite film with a large grain size for the enhanced photovoltaic performance of PSCs [18]. Particularly, methylammonium iodide is found to play a key role in regulating the crystalline rate of the MAPbI_3_ film. The formation of the weak hydrogen bond between additives and the MAI is possibly an effective route to control the growth rate of the perovskite film. Unfortunately, designing such additives still exhibits a formidable challenge.

**Figure 1:**
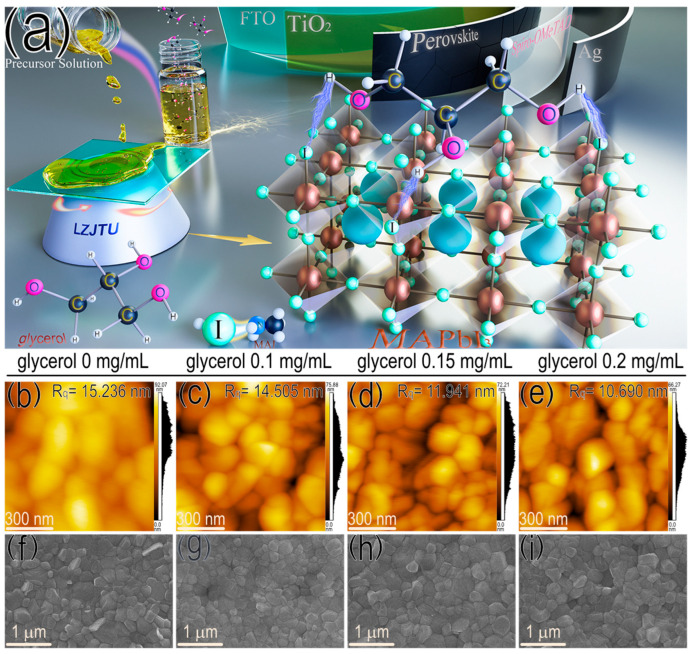
(**a**) Schematic illustration of modulating the crystal formation process of perovskite films with glycerol. (**b**–**e**) AFM and (**f**–**i**) top-view SEM images of perovskite films doped without and with different amounts of glycerol.

Herein, a glycerol with three hydroxyl groups was employed to modulate the crystal formation process of MAPbI_3_ film, as depicted in Figure 1a. A weak hydrogen bond involving iodide (-H-I-) was formed between the hydrogen atom in the hydroxyl group of glycerol and the iodide atom in MAI. Doping glycerol into the MAPbI_3_ film not only increased the grain size and carrier transport efficiency of the film, but also effectively decreased its surface defect and nonradiative recombination of carriers. Finally, the champion PCE of up to 16.84% for glycerol-doped PSCs was obtained compared with that of the control group (14.33%), corresponding to the improved parameters, including open-circuit voltage (*V_oc_*), short-circuit current density (*J_sc_*) and fill factor (*FF*) from 1.041 to 1.093 V, 22.76 to 23.14 mA/cm^2^ and 60.45 to 66.59%, respectively. Moreover, the target device presented excellent storage stability for up to 30 days with 92.05% of initial PCE, compared with 83.57% (only 24 days) for the pristine device.

## 2. Experimental Section

### 2.1. Experimental Materials and Reagents

Isopropyl titanate (C_12_H_28_O_4_Ti, 97.0%), acetonitrile (ACN, 99.9%) and chlorobenzene (CB, 99.5%) were purchased from Sigma Aldrich, St. Louis, MO, USA. Isopropyl alcohol (IPA, 99.9%), N, N-dimethylformamide (DMF, 99.9%) and dimethyl sulfoxide (DMSO, 99.8%) were obtained from Shanghai Aladdin Biochemical Technology Co., Ltd, Shanghai, China. Methylammonium iodide (MAI, 99.5%) was purchased from Xi’an Yuri Solar Co., Ltd, Xi’an, China. Lead iodide (PbI_2_, 99.9%), Lithium bis(trifluoromethanesulphonyl) imide (Li-TFSI, 99.95%), 4-tert-butylpyridine (4-tBP, 96.0%) and 2, 2′, 7, 7′-tetrakis-(N, N-di-p-methoxyphenylamine)-9, 9′-spirobifluorene (Spiro-OMeTAD, 99.86%) were purchased from Advanced Election Technology Co., Ltd, Yingkou, China. Glycerol (99.0%) was purchased from Sichuan Xilong Science Co., Ltd, Chengdu, China. Fluorine-doped tin oxide (FTO, 14 Ω, 1.5 × 1.5 cm^2^) was purchased from Advanced Election Technology Co., Ltd, Yingkou, China. All chemicals were used as received, without a further purification process.

### 2.2. Perovskite Solar Cells Fabrication

In this paper, the PSC with the n-i-p planar structure of FTO/TiO_2_/MAPbI_3_/Spiro-OMeTAD/Ag was fabricated, in which TiO_2_, MAPbI_3_, Spiro-OMeTAD and Ag present an electron transport layer (ETL), perovskite active layer, hole transport layer (HTL) and top electrode, respectively. FTO substrates were cleaned with detergent solutions, acetone, isopropyl alcohol and deionized water by an ultrasonic cleaning device (Skymen Cleaning Equipment Shenzhen Co., Ltd, Shenzhen, China) for 20 min, respectively. For dense TiO_2_ layer fabrication, 40 μL isopropyl titanate solution (0.43 mmol/mL) was spin-coated on a cleaned FTO substrate at 2500 rpm for 20 s. Then, the substrate was annealed at 120 °C for 10 min in a N_2_ atmosphere glove box before being calcined at 500 °C for 30 min in a muffle furnace to obtain the dense TiO_2_ layer. Anti-solvent engineering was then adopted to fabricate the MAPbI_3_ layer. For the preparation of the glycerol solution, different amounts (0, 10, 15, 20, 25 mg) of glycerol were added into 1 mL of a mixed solution containing 0.8 mL N-dimethylformamide (DMF) and 0.2 mL dimethylsulfoxide (DMSO). Then, these stock solutions were followed by stirring overnight in a N_2_ glovebox at room temperature. After that, these solutions were diluted 100 times, using the mixed solution (DMF/DMSO = 4:1, *v*/*v*) to keep the concentration of glycerol at 0, 0.1, 0.15 and 0.2 mg/mL (denoted as “solution 1”). Then, 1.1525 g PbI_2_ and 0.397 g MAI (molar ratio of 1:1) were mixed in the 2 mL “solution 1” before stirring overnight at room temperature in a nitrogen atmosphere. In addition, 35 μL perovskite precursor solution was then dropped onto the TiO_2_ layer and followed by a two-step spin-coating, in which the first step started at 1000 rpm for 3 s and the second step began at 4000 rpm for 50 s. Then, 200 μL anhydrous chlorobenzene as an anti-solvent was dripped onto the substrate at the fifth second during the second step at a dripping rate of 200 μL/S and was followed by annealing at 100 °C for 10 min. Then, the precursor solution of the hole transport layer was prepared by mixing 1 mL Spiro-OMeTAD (72.3 mg/mL), 17.5 μL Li-TFSI (520 mg/mL) and 28.8 μL tBP. Then, 20 μL of the above solution was deposited onto the perovskite film at a parameter of 5000 rpm for 30 s and followed by oxidation in a desiccator overnight. Finally, a Ag top electrode (~100 nm) was deposited onto the Spiro-OMeTAD layer under a vacuum pressure (6 × 10^−3^ Pa) and a current of 40 A for 20 min.

### 2.3. Material Characterization

X-ray diffraction (XRD) analysis was performed by using the X-ray diffractometer (Rigaku, Kyoto, Japan), with a scanning speed of 10 degrees min^−1^, step size of 0.026 degrees and scanning range from 10 to 60 degrees. Scanning electron microscope (SEM) images were acquired, using the SU8020 device (Shimadzu, Kyoto, Japan). Ultraviolet-visible absorption spectra were recorded on the UV-1800 spectrometer (Shimadzu, Kyoto, Japan) with a scanning wavelength range from 500 to 1100 nm. A fluorescence spectrophotometer (F-7100, Hitachi, Tokyo, Japan) was used to investigate the photoluminescence intensity of perovskite films with an excitation wavelength of 460 nm, and the corresponding emission wavelength range was set from 650 to 850 nm. Ultraviolet photoelectron spectroscopy (UPS) measurements were carried out using an ESCALAB XI+ device (ThermoFisher Scientific, Waltham, MA, USA).

### 2.4. Device Characterization

The *J-V* characteristics of PSCs were investigated using an AM 1.5 G solar simulator (XEC-300 M2, San-Ei Electric Co., Ltd. Osaka, Japan) and Sourcemeter (2400, Keithley, Beaverton, OR, USA). Device measurements, including *J-V* curves under light or dark irradiation, space-charge limited current (SCLC) and light-intensity-dependent *V_oc_*, were performed in a glove box with an incident light power density of 100 mW/cm^2^, an effective testing area of 0.057 cm^2^ and a testing voltage ranging from −0.2 to 1.2 V. External quantum efficiency (EQE) measurement was performed with a commercial EQE/incident photon to charge carrier efficiency (IPCE) setup (7-SCSpec IPCE, Beijing 7-Star Optical Instruments Co., Ltd, Beijing, China), in which an integrated standard single-crystal Si photovoltaic cell (S1337-101BQ, SOFN instruments Co., Ltd., Beijing, China, calibrated by National Institute of Metrology of China) was equipped to calibrate the light intensity at each wavelength. The scanning range for IPCE analysis was set from 300 to 800 nm. The photoelectronic measurements of PSCs were conducted using an electrochemical workstation (CHI 600D, Hua Chen, Shanghai, China). For electrochemical impedance spectroscopy (EIS) analysis, the initial voltage was set at 0.8 V, 1 × 10^6^ Hz for high frequency and 1 Hz for low frequency.

## 3. Results and Discussion

### 3.1. Modulating the Perovskite Film with Glycerol

The glycerol molecule with a chemical formula of C_3_H_8_O_3_ contains three hydrogen groups, is colorless and has a high viscosity of up to 1412 mPa S at 20 °C (Figure S1) [19]. Generally, glycerol is a kind of water-soluble liquid and can be dissolved in a mixed solution of DMF/DMSO. In Figure S2a, a transparent solution is observed for the MAPbI_3_ solutions, both with and without glycerol doping. There are no visible precipitations for these solutions, with ascending amounts of glycerol from 0 to 0.2 mg/mL. The UV-vis spectra of these solutions in Figure S2b indicate the similar optical absorption intensities and identical profiles for the precursor solutions, with and without glycerol. Therefore, the glycerol is an appropriate additive to modulate the nucleation and growth of the MAPbI_3_ film by dissolving it in the precursor solution. As a result, the MAPbI_3_ film doped with glycerol exhibits a decreased root mean square roughness (R_q_) value from 15.236 to 10.690 nm (Figure 1b–e) and an increased contact angle from 54.5° to 63.7° (Figure S3), as demonstrated by the atomic force microscope (AFM) and contact angle (CA) results. The decreased surface roughness of the MAPbI_3_ film doped with glycerol suggests better interface contact between the active layer and upper hole transporting layer (HTL) for enhanced hole-transporting efficiency. In addition, from the top-view scanning electron microscope (SEM) images in Figure 1f–i, MAPbI_3_ films are obtained with a uniform surface coverage and enlarged grain size (from 245.10 ± 78.89 to 315.15 ± 78.49 nm in Figure S4) after doping with increased amounts of glycerol from 0.1 to 0.15 mg/mL. However, excessive glycerol (0.2 mg/mL) exhibits a weak effect on the grain size of the MAPbI_3_ film, indicating that 0.15 mg/mL of glycerol is optimal to obtain the MAPbI_3_ film with a larger grain size. X-ray diffraction (XRD) analysis also confirms this result, as demonstrated by the improved peak intensities of perovskite film with and without the glycerol doping at both 14.6° and 29.03° (corresponding to (110) and (220) crystal plane) in Figure S5 [20,21]. Therefore, glycerol doping contributes to an enhanced crystallinity of MAPbI_3_-based film.

**Figure 2:**
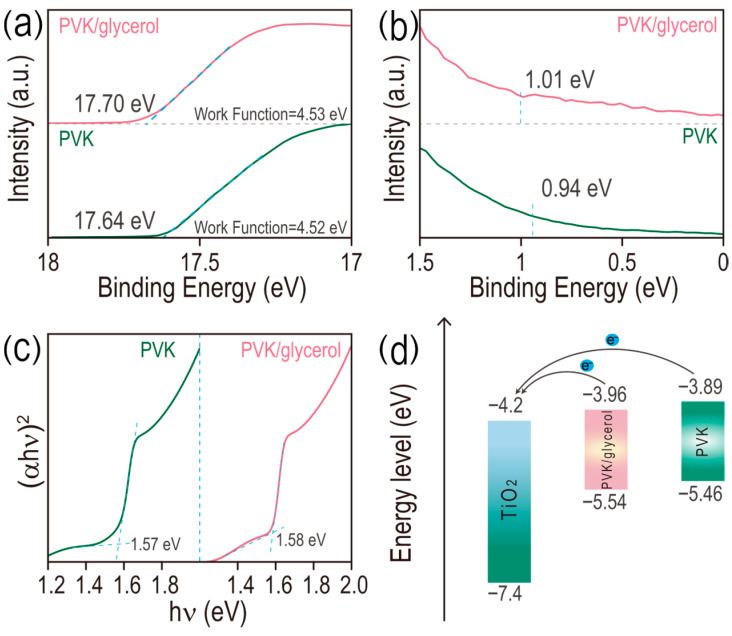
(**a**) UPS spectra of the secondary electron cut-off region (*E_cut_*), (**b**) the Fermi edge (*E_edge_*) and (**c**) Tauc plots of perovskite films, doped with and without glycerol (0.15 mg/mL). (**d**) The energy-level diagram of both perovskite and perovskite/glycerol films in PSCs.

Enhanced crystallization of MAPbI_3_ film treated with glycerol possibly has positive effects on its optical properties. Identical optical absorption profiles are observed for perovskite films with and without glycerol doping (Figure S6a), indicating negligible effects of glycerol doping on the band gap of the MAPbI_3_ film. Especially for glycerol doping (0.15 mg/mL), a slight enhancement of optical absorption in the whole range is observed and its intensity at 750 nm is 1.16 times higher than that of pristine perovskite film (Figure S6b), which is possibly caused by the improved crystallinity of MAPbI_3_ film doped with glycerol. The increased optical absorption capability of MAPbI_3_ film is conducive to harvesting more photons for the enhanced photovoltaic performance of PSCs. The transporting efficiency of photogenerated carriers strongly depends on the photoluminescence intensity of MAPbI_3_-based perovskite film. In Figure S7, the maximum emission wavelength for both the pristine and glycerol-treated films is located at 776 nm. Noticeably, the emission intensity of 776 nm for the glycerol-treated film is 1.41 times that of the pristine film, demonstrating the decreased defect density and suppressed nonradiative recombination of the MAPbI_3_ film doped with glycerol. Ultraviolet photoelectron spectroscopy (UPS) results of both the pristine and glycerol (0.15 mg/mL)-doped perovskite films coated onto a glass substrate are provided in Figure 2a,b. The cut-off binding energy (*E*_cut_) of perovskite and perovskite/glycerol are 17.64 and 17.70 eV, respectively. Then, the calculated work function (*WF*) of the two films is 4.52 and 4.53 eV, based on the equation of *WF =* 21.22 − (*E*_cut_ − *E*_edge_), where 21.22 eV presents the emission energy of the helium irradiation. From the Fermi edge (*E*_edge_) results obtained from Figure 2b (0.94 and 1.01 eV indicating the *E*_edge_ of the pristine perovskite and perovskite/glycerol films, respectively), the conduction bands (*E*_CB_) of the two films are calculated to be 3.89 and 3.96 eV for the perovskite and perovskite/glycerol films, respectively [22,23,24]. In addition, similar band gaps are obtained for the two films (1.57 eV and 1.58 eV for perovskite and perovskite/glycerol, respectively) from the Tauc plots in Figure 2c. The conductive band, valence band and corresponding band gaps of the films are summarized in Table S1 and the related energy-level diagram is depicted in Figure 2d. The conduction band of perovskite/glycerol (0.15 mg/mL) is much closer to that of the TiO_2_ (electron transport layer) compared with the pristine perovskite film, thereby enhancing the interface energy band alignment and benefiting the electron transport from MAPbI_3_ to the electron transport layer (ETL), which will contribute to the enhancement of the open circuit voltage (*V*_oc_) of PSCs.

### 3.2. Formation of Hydrogen Bond Involving Iodide

**Figure 3:**
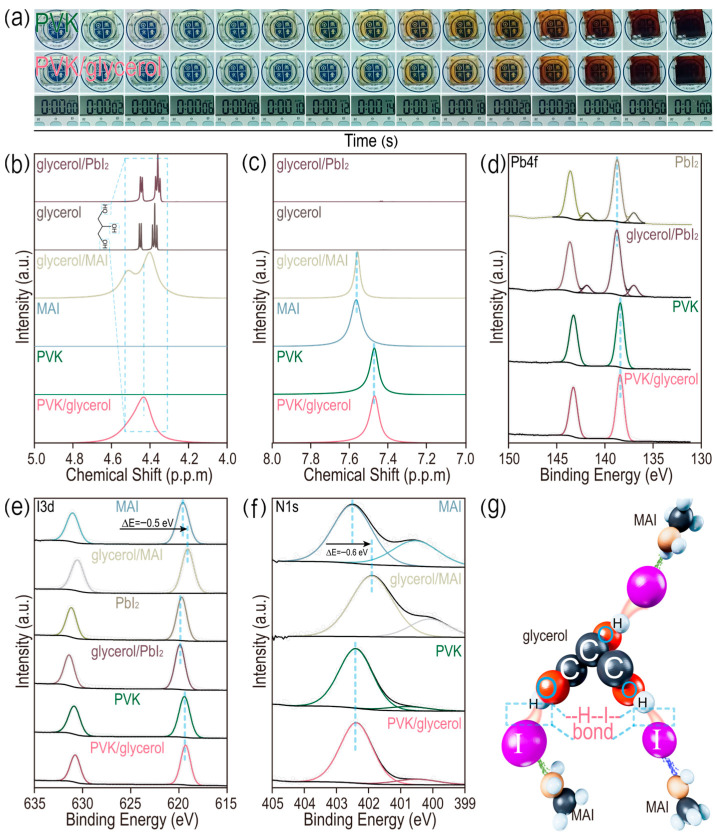
(**a**) Photographs of growth process of perovskite films doped with and without glycerol. Magnified ^1^H-NMR spectra of (**b**) hydroxyl groups and (**c**) -NH_2_ in MAI. High-resolution XPS of (**d**) Pb 4f, (**e**) I 3d and (**f**) N 1s. (**g**) Schematic illustration of weak hydrogen bond involving iodide (-H-I-) between hydrogen atoms in glycerol and iodide atom in MAI.

It is vital to give a deep insight into modulating the crystallinity of perovskite films through glycerol doping. The detailed growth processes of films doped with and without glycerol are recorded in Figure 3a. The pristine perovskite film turns a light brown color after 10 s of annealing treatment at 100 °C. However, the same phenomenon occurs after 14 s for the film doped with the glycerol (0.15 mg/mL), indicating the time delay of the crystal growth of the perovskite film modulated by the glycerol. Then, liquid-state ^1^H magnetic resonance (^1^H-NMR) is employed to analyze the chemical interactions between glycerol and methylammonium iodide (MAI) or lead iodide (PbI_2_) in a dimethyl sulfoxide (DMSO-d_6_) solvent. Full survey spectra of samples are given (Figure S8a), in which tetramethylsilane (TMS) is used for calibration. In Figure 3b, dual resonance signals between 4.2 and 4.6 ppm belong to the hydroxyl groups (-OH) of glycerol for samples including glycerol and glycerol/PbI_2_. There is a negligible difference for the peaks of hydroxyl groups between the glycerol and glycerol/PbI_2_. However, the peak widths of the hydroxyl groups are increased and no chemical shift is detected for both the glycerol/MAI and glycerol/PVK, possibly indicating the formation of a weak chemical interaction, rather than a strong chemical bond (such as ionic bonds and covalent bonds) between the hydroxyl group of glycerol and MAI [25,26,27]. In Figure 3c, peaks in the range from 7.4 to 7.8 ppm correspond to the amino hydrogen atoms (-NH_2_) of methylammonium iodide. Negligible peak shifts are demonstrated for the glycerol/MAI and pristine MAI (7.562 vs. 7.569 ppm, respectively). An identical result also occurs for both the perovskite and perovskite/glycerol solution (7.475 vs. 7.474 ppm, respectively). In Figure S8b, peaks in the range from 2.0 to 2.6 ppm can be attributed to the methyl group of MAI. The above ^1^H-NMR results provide solid evidence for the presence of a weak chemical interaction between the hydroxyl group of glycerol and a chemical group of MAI, except from the -NH_2_ and -CH_3_ groups [28,29,30,31,32,33]. Then, high-resolution X-ray photoelectron spectra (XPS) analysis is adopted to disclose this group in MAI. There is negligible interaction between glycerol and Pb^2+^ for samples of both the PbI_2_ and MAPbI_3_ with and without glycerol in Figure 3d. However, both I 3d and N 1s patterns of MAI shift toward a lower binding energy, suggesting that both iodide and nitrogen atoms of MAI accept electrons from glycerol in Figure 3e,f. It is noted that glycerol-doping exhibits a weak influence on the PbI_2_ and perovskite in Figure 3e,f. Owing to the relatively lower electronegativity of iodide (2.66) than both oxygen (3.44) and nitrogen (3.04), iodide exhibits a weak capability of accepting electrons from the hydroxyl group of glycerol to generate the strong hydrogen bond. A combination of ^1^H-NMR and XPS results suggest the existence of a weak hydrogen bond involving iodide (-H-I-) between the hydrogen atoms of glycerol and an iodide atom of a MAI molecule, in which iodide ions accept electrons from hydrogen ions, as depicted in Figure 3g. The formed weak -H-I- bond in the glycerol/MAI is responsible for slowing down the doping rate of the MAI into the center of PbI_2_ with a cubic crystal structure to form MAPbI_3_, resulting in the relative low crystallization rate of perovskite film with a large grain size and dense surface coverage for high-performance PSCs.

### 3.3. Photovoltaic Performance of Perovskite Solar Cells

**Figure 4:**
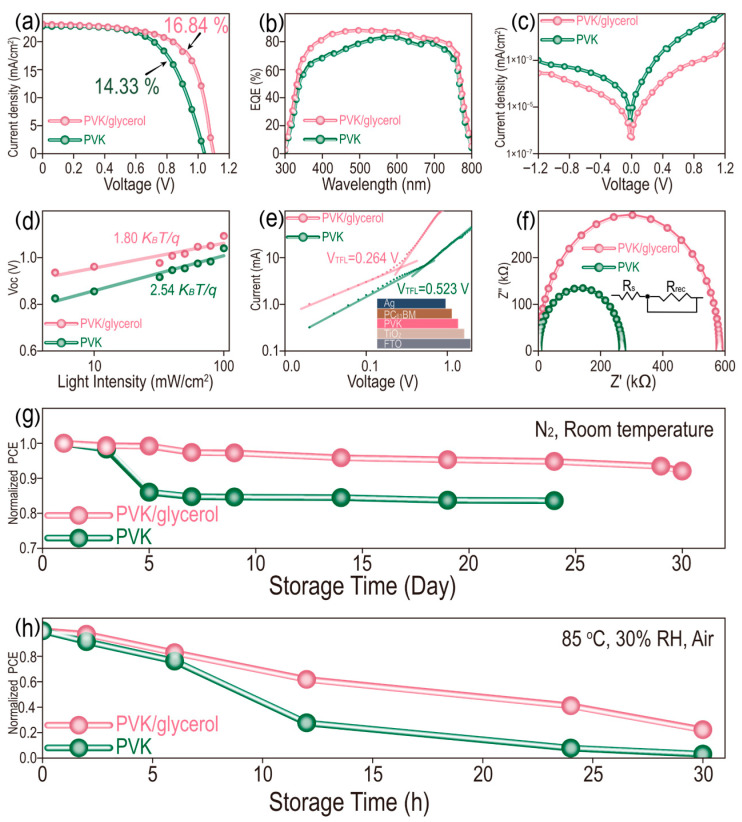
(**a**) *J-V* curves under light condition, (**b**) EQE, (**c**) *J-V* curves under dark condition, (**d**) light intensity-dependent *V_OC_*, (**e**) EIS, (**f**) SCLC and long-term working stability of PSCs at (**g**) N_2_/room temperature and (**h**) 85 °C/30% RH in air.

Generally, perovskite films with improved crystallinity result in the increased photovoltaic performance of PSCs. It is crucial to investigate the influence of glycerol-doping on the photovoltaic performance of PSCs. Therefore, PSCs with a structure of FTO/TiO_2_/MAPbI_3_/Spiro-OMeTAD/Ag are fabricated, in which MAPbI_3_ is doped with or without glycerol (0.15 mg/mL). Cross-sectional SEM images of PSCs with and without glycerol are compared in Figure S9. A similar internal growth of perovskite films occurs in all PSCs with dense contact between the active layer (MAPbI_3_) and ETL (TiO_2_) or HTL (Spiro-OMeTAD), demonstrating that glycerol doping does not affect the structure of PSCs. Then, the photovoltaic performance of the two films is investigated by the *J-V* curves in Figure 4a and the corresponding photovoltaic parameters, including open-circuit voltage (*V_OC_*), short-circuit current density (*J_SC_*), fill factor (*FF*) and power conversion efficiency (PCE) are listed in Table S2. A champion PCE reaches up to 16.84% for devices treated with glycerol (0.15 mg/mL), which is an improvement of 17.51% compared with the pristine device (PCE of 14.33%). Similarly, *J_SC_*, *V_OC_* and *FF* also increase for glycerol doping compared to the control group. The enhanced PCE is mainly attributed to the improved *V_OC_* and *FF* for glycerol-doped devices with better energy-level matching and decreased surface defects. In addition, it is necessary to investigate the hysteresis effect of PSCs to disclose the defects and ion migration inside the device, which can influence its long-term stability and service life. In Figure S10, the device doped with glycerol exhibits a relatively small hysteresis, as demonstrated by the reverse scanning (RS) and forward scanning (FS) of PCE, for which the values are 16.84% and 14.48%, respectively. In contrast, the pristine device has relatively significant hysteresis, with 14.33% and 9.53% of PCE for RS and FS, respectively. The hysteresis index (HI), based on the formula of HI = (PCE_reverse_ − PCE_forward_)/PCE_reverse_, is calculated to be 0.140 and 0.335 for the glycerol-modified and pristine devices, respectively. The decreased HI indicates less defects and outstanding long-term working stability of PSCs [34,35,36]. Subsequently, twenty independent PSCs, with and without glycerol doping, are fabricated to investigate their repeatability to exclude the factor of experimental coincidence, and the statistical box charts of photovoltaic parameters are shown in Figure S11. Similar results are obtained for the glycerol-doped and pristine devices. The average PCE of the glycerol-doping group is 15.10%, which is higher than that of the pristine device (13.36%). Generally, the defects and the carrier recombination in perovskite films have negative effects on their photovoltaic efficiency [37,38]. In Figure 4b, the external quantum efficiency (EQE) spectral response of a device doped with glycerol exhibits a more remarkable enhancement than that of the pristine device in a range from 300 to 800 nm, which manifests the faster carrier transport and higher extraction capability of PSCs caused by the reduced internal defects in perovskite film after glycerol doping. Particularly, an obvious enhancement of EQE in range from 300 to 550 nm can be attributed to the increased optical absorption of MAPbI_3_ film doped with glycerol in a range from 300 to 600 nm in Figure S12. For the dark *J-V* curves in Figure 4c, the device doped with glycerol presents a decreased current density, with 3.06 × 10^−5^ mA/cm^2^ compared with 1.39 × 10^−4^ mA/cm^2^ for the control device at their lowest point, suggesting a lower leakage current and reduced nonradiative recombination, which can be attributed to the dense interface contact generated on each layer of the device. Furthermore, the nonradiative recombination efficiency in these devices can be illustrated by the light-intensity-dependent *V_oc_* measurement in Figure 4d. The ideal factors (n) of PSCs decrease from 2.54 to 1.80 after glycerol doping, based on the following formula: *V_oc_*= *nK_B_Tln*(*P_light_*)/*q*, where *K_B_* is the Boltzmann constant, *T* is the Kelvin temperature, *P_light_* is the light intensity and q is the elementary charge [39,40]. The lower *n* value for PSCs doped with glycerol demonstrates the effective suppression of nonradiative recombination, leading to the reduction in *V_oc_* loss and leakage current in PSCs. The internal charge transfer behaviors of the two devices under dark conditions are then investigated in Figure 4e by the Nyquist plots and fitting equivalent circuit diagram. The series resistance (*R_s_*) represents the *V_oc_* loss caused by the material resistance and contact resistance. A larger semicircular diameter means a higher recombination resistance (*R_rec_*) and corresponding lower probability of nonradiative recombination. As a result, the PSC doped with glycerol exhibits a lower *R_s_* and higher *R_rec_* (Table S3), which indicate the increased *V_oc_* and reduced nonradiative recombination. In addition, the defect state density of the perovskite film can be quantitatively evaluated by space charge limited current (SCLC) analysis, using an electron-only device with the structure of FTO/TiO_2_/MAPbI_3_/PC_61_BM/Ag. The trap density (*N_trap_*) can be calculated based on the following formula: *N_trap_* = 2*ε*_0_*ε_r_V_TFL_*/*eL*^2^, where *V_TFL_*, *ε*_0_, *ε_r_*, *e* and *L* represent trap-filled limit voltage from SCLC curve fitting, vacuum permittivity (8.854 × 10^−12^ F/m), relative dielectric constant of MAPbI_3_ (28.8), elementary charge (1.602 × 10^−19^ C) and thickness of perovskite film (356.5 and 356.0 nm for MAPbI_3_ and MAPbI_3_/glycerol (0.15 mg/mL)), respectively. In this calculation formula, the smaller *V_TFL_* means a lower *N_trap_* value, resulting in decreased defects in the device [41,42]. In Figure 4f, the *V_TFL_* of PSCs with and without glycerol doping are determined to be 0.264 and 0.523 V, respectively. Thus, the corresponding *N_trap_* is calculated to be 6.63 × 10^15^ and 1.31 × 10^16^ cm^−3^ for the device doped with and without glycerol, respectively. The long-term stability of PSCs is one of the main factors considered in the practical production process. After 30 days of storage of PSCs in a nitrogen atmosphere, the glycerol-doped device keeps 92.05% of the initial PCE, compared with only 83.57% for the pristine device in Figure 4g. Unfortunately, the pristine device suffers from deterioration after storage for 24 days. In addition, these PSCs are further subjected to evaluating their working stability under a condition of 85 °C and 30% RH in air (Figure 4h). The PSC treated with glycerol still maintains 22.28% of the pristine PCE, which is higher than the 3.04% of PCE for the control PSC after 30 h of treatment. The above results indicate that glycerol doping delays the growth of perovskite film, with fewer internal and surface defects towards high-performance PSCs, as demonstrated by the increased charge transport and inhibited carrier recombination [43].

## 4. Conclusions

In summary, a novel strategy is designed to delay the growth process of MAPbI_3_ films by incorporating glycerol into the perovskite precursor solution, which forms a hydrogen bond involving iodide between hydrogen atom of the hydroxyl group in glycerol and the iodide atom of MAI. Glycerol doping improves the crystallinity of perovskite film with a large grain size and uniform surface coverage. In addition, glycerol doping is also capable of suppressing the nonradiative recombination and modulating the energy-level arrangement between the MAPbI_3_ layer and the ETL for an improved electron transporting efficiency. As a result, glycerol doping increases the yield of PCE from 14.33% to 16.84% and obtains satisfactory stability for up to 30 days with 92.05% of initial PCE, compared with only 83.57% for the pristine PSCs. This finding explores the potential of the hydrogen bond involving iodide in modulating the growth of perovskite film for high-performance and stable PSCs.

## Data Availability

The data that support this study is available from the corresponding author upon reasonable request.

## Supplementary Materials

The following supporting information can be downloaded at: https://www.mdpi.com/article/10.3390/mi17010015/s1, Figure S1. Molecular structure of glycerol. Figure S2. (a) photographs and (b) corresponding UV-vis spectra of precursor solution for preparing perovskite film containing increased amounts of glycerol. Figure S3. Contact angle of MAPbI3 film with and without glycerol treatment. Figure S4. Average grain size distributions of perovskite films doped with different amounts of glycerol. Figure S5. (a) XRD patterns and corresponding peak intensities at (b) 14.6o and (c) 29.03o of MAPbI3 films after incorporating with increased amounts of glycerol. Figure S6. (a) UV-vis spectra of MAPbI3 films treated with increased amounts of glycerol and corresponding absorption intensities at 750 nm. Figure S7. Photoluminescence spectra of MAPbI3 film coated onto glass substrate with excitation wavelength at 460 nm. Figure S8. Liquid-state 1H magnetic resonance (1H-NMR) spectra of samples dissolved in dimethyl sulfoxide (DMSO-d6) with (a) survey (−1–10 ppm) and (b) magnification (2.0–2.6 ppm) of scale. Figure S9. Cross sectional SEM images of PSCs with structure of FTO/TiO_2_/MAPbI3/Spiro-OMeTAD/Ag in which MAPbI3 is doped (a) with and (b) without glycerol (0.15 mg/mL). Figure S10. Hysteresis effects of (a) pristine and (b) glycerol doped PSCs. Figure S11. Statistical results of J-V parameters including (a) PCE, (b) Jsc, (c) FF and (d) Voc of twenty PSCs doped with and without glycerol. Figure S12. UV-vis spectra of MAPbI3 film with and without glycerol in range from 300 to 600 nm. Table S1. The parameters of perovskite films with and without glycerol extracted from the XPS spectra and Tauc plot. Table S2. The photovoltaic parameters for glycerol-doped (0.15 mg/mL) and pristine devices. Table S3. Fitted Rs and Rrec data of EIS curves of PSCs incorporated with and without glycerol.
