# Supplementary material for: Weak Hydrogen Bond with Iodide Modulating Crystallization of Methylammonium Lead Iodide for High-Performance Perovskite Solar Cells
Source: Micromachines (Basel). 2025 Dec 24;17(1):15. doi: 10.3390/mi17010015 (PMC12843685; doi:10.3390/mi17010015)
Supplement: Supplementary file 1 [file micromachines-17-00015-s001.zip › micromachines-4029751-supplementary.pdf]

Supporting information

Weak Hydrogen Bond with Iodide Modulating Crystallization of Methylammonium Lead Iodide for High-Performance Perovskite Solar Cells

Ning Kang[1,*], Lu Li[1], Zhe Wan[1], Liping Yang[1], Zhen Liang[1], Li Chen[1], Peng Li[1], Yongrong Sun[2], Zuyong Wang[3] and Chenglong Wang[1,*]

1 National Engineering Research Center for Technology and Equipment of Environmental Deposition, Solar Thermal Industry Research Institute of Gansu Province, Lanzhou Jiaotong University, Lanzhou 730070, China

2 Guang Dong Engineering Technology Research Center of Biomaterials, Institute of Biological and Medical Engineering, Guangdong Academy of Sciences, Guangzhou 510316, China

3 College of Materials Science and Engineering, Hunan University, Changsha 410082, China

Supplemental Figures and Tables

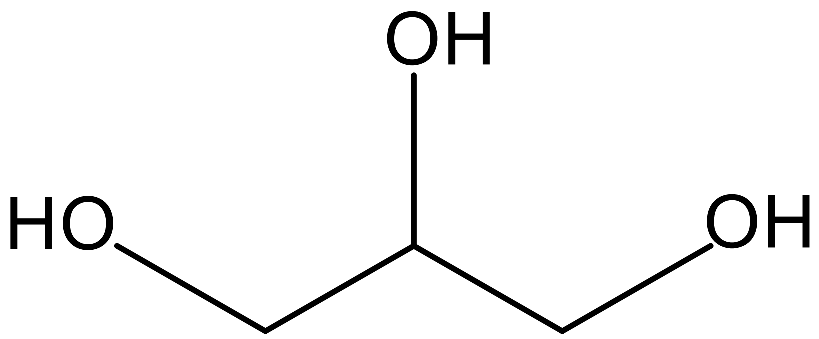

Figure S1. Molecular structure of glycerol.

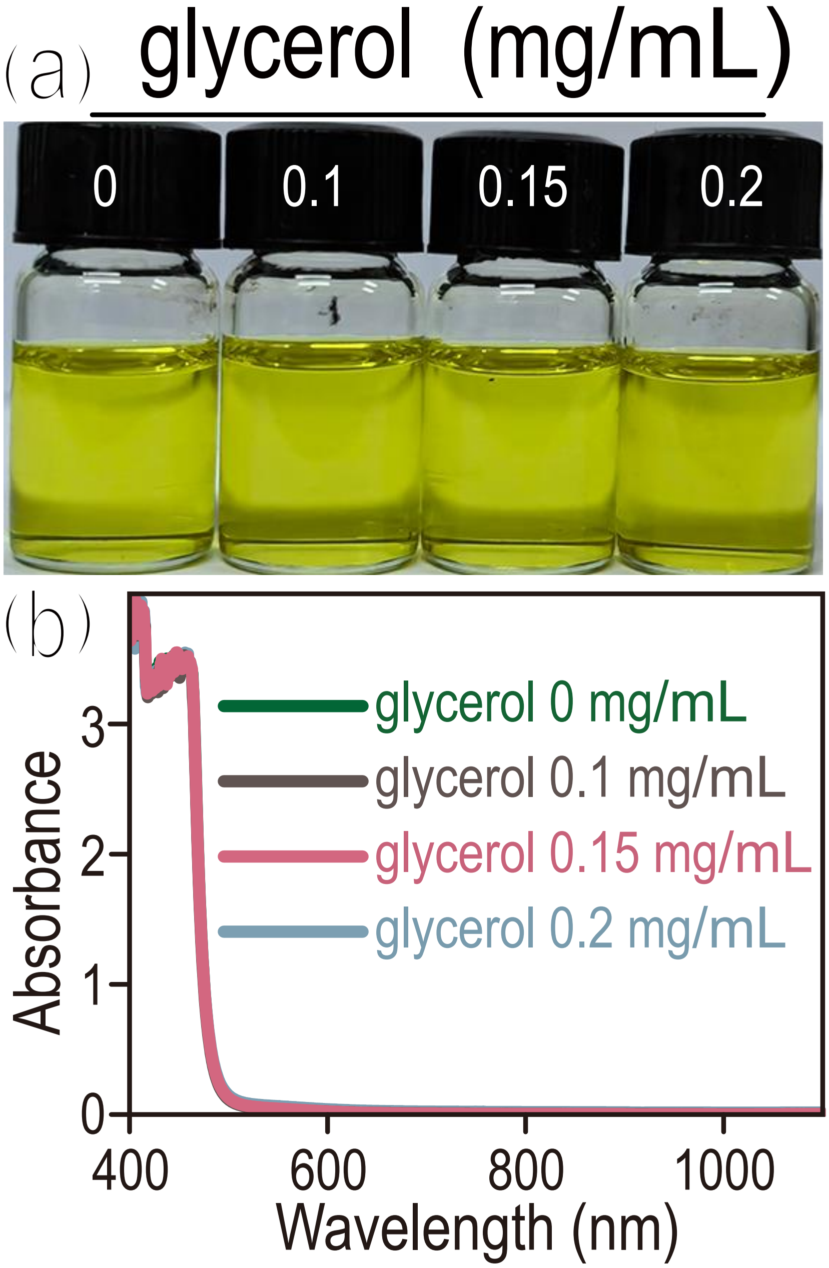

Figure S2. (a) photographs and (b) corresponding UV-vis spectra of precursor solution for preparing perovskite film containing increased amounts of glycerol.

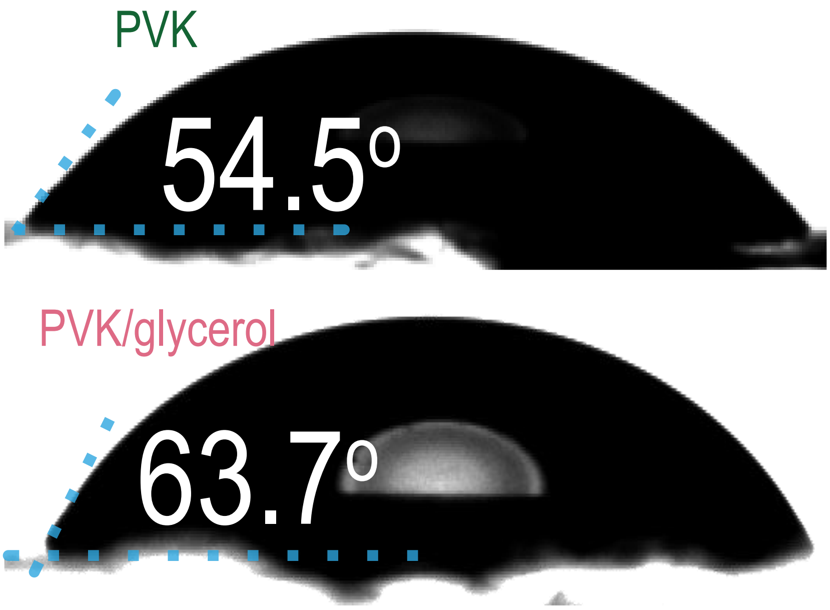

Figure S3. Contact angle of $MAPbI_3$ film with and without glycerol treatment.

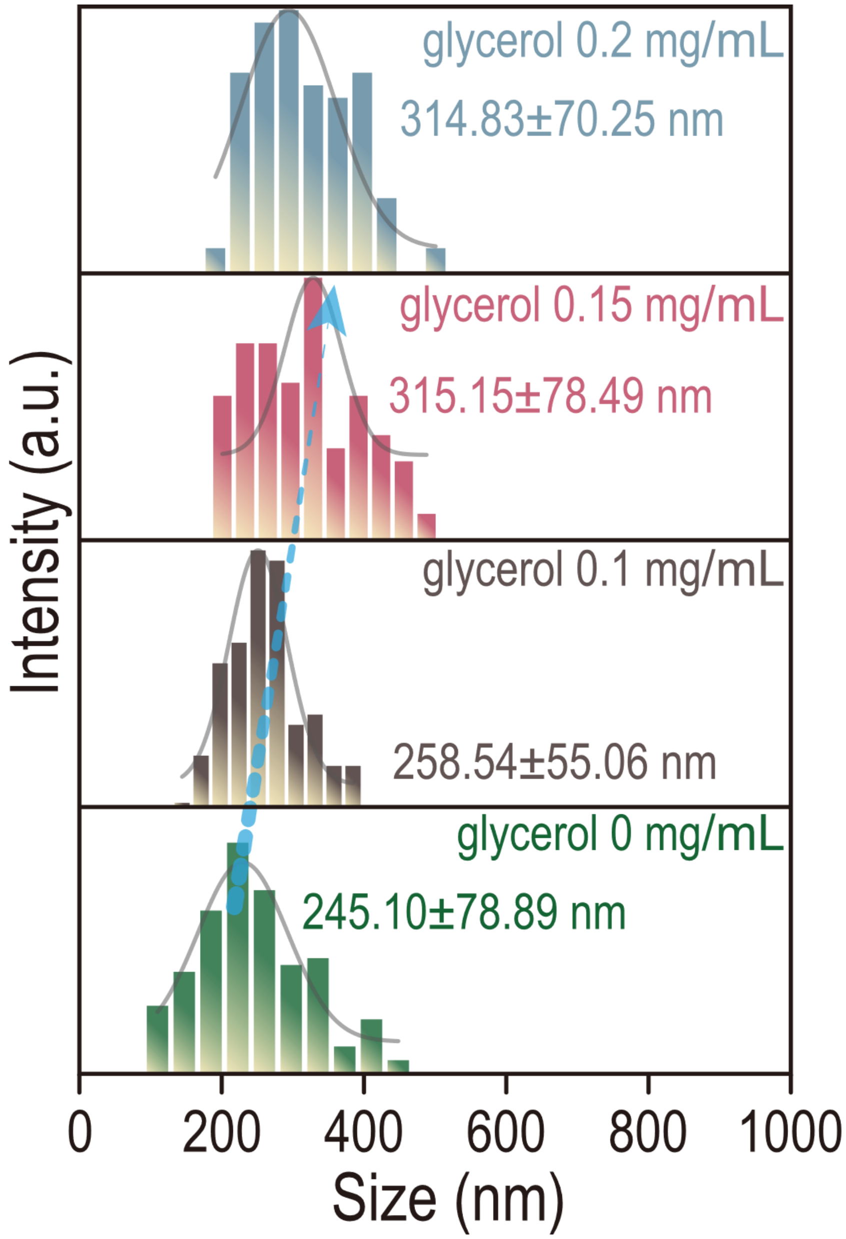

Figure S4. Average grain size distributions of perovskite films doped with different amounts of glycerol.

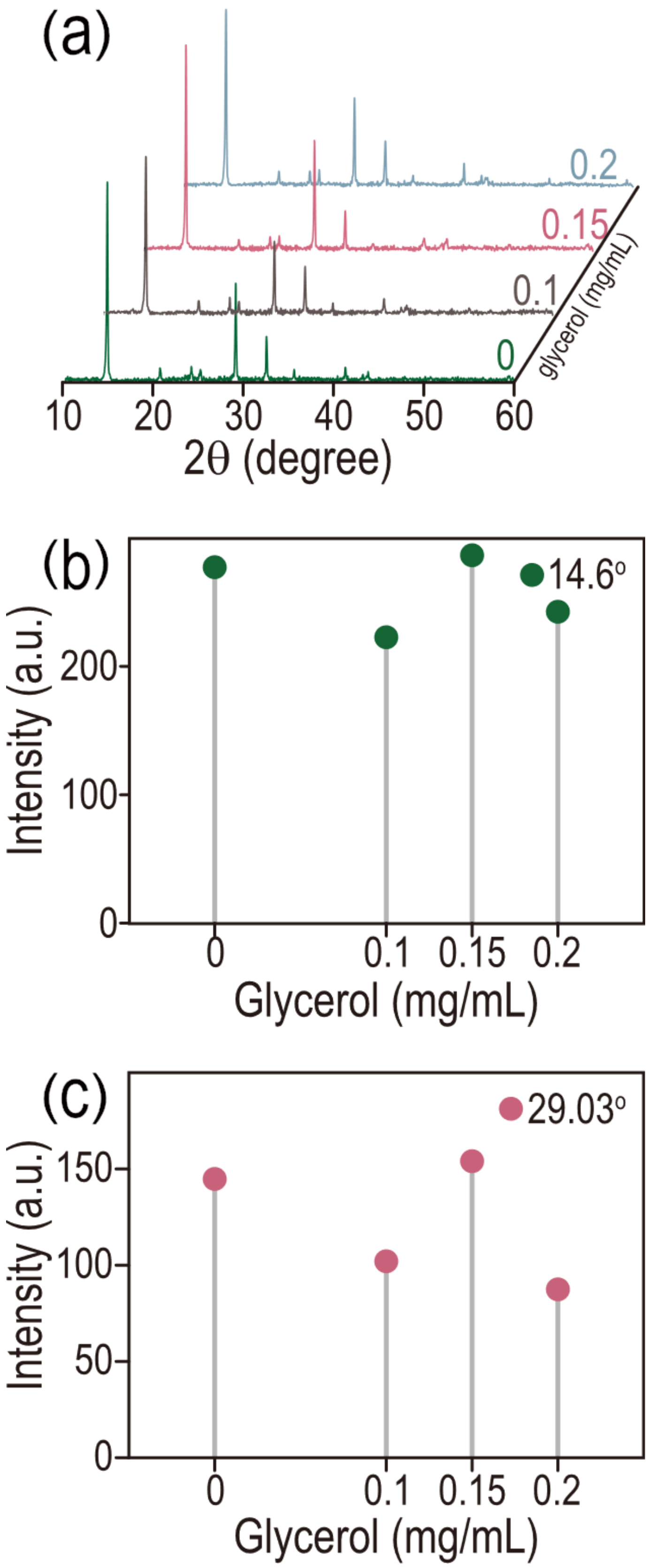

Figure S5. (a) XRD patterns and corresponding peak intensities at (b) 14.6° and (c) 29.03° of $MAPbI_3$ films after incorporating with increased amounts of glycerol.

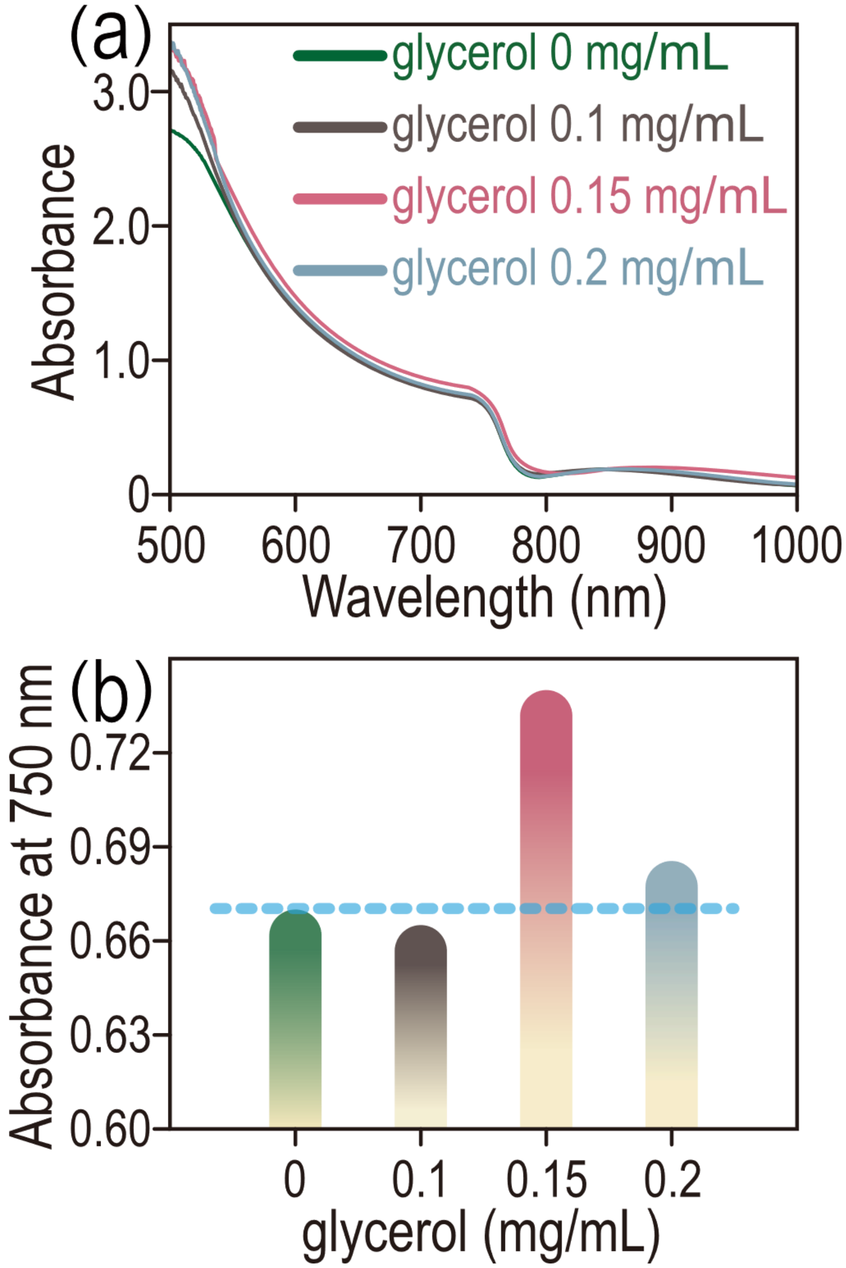

Figure S6. (a) UV-vis spectra of $MAPbI_3$ films treated with increased amounts of glycerol and corresponding absorption intensities at 750 nm.

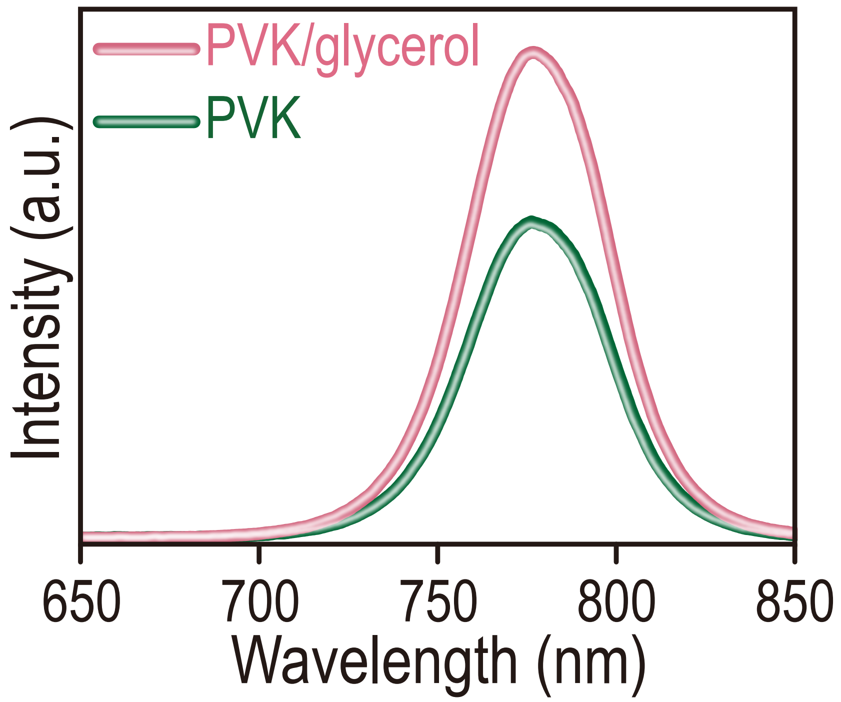

Figure S7. Photoluminescence spectra of $MAPbI_3$ film coated onto glass substrate with excitation wavelength at 460 nm.

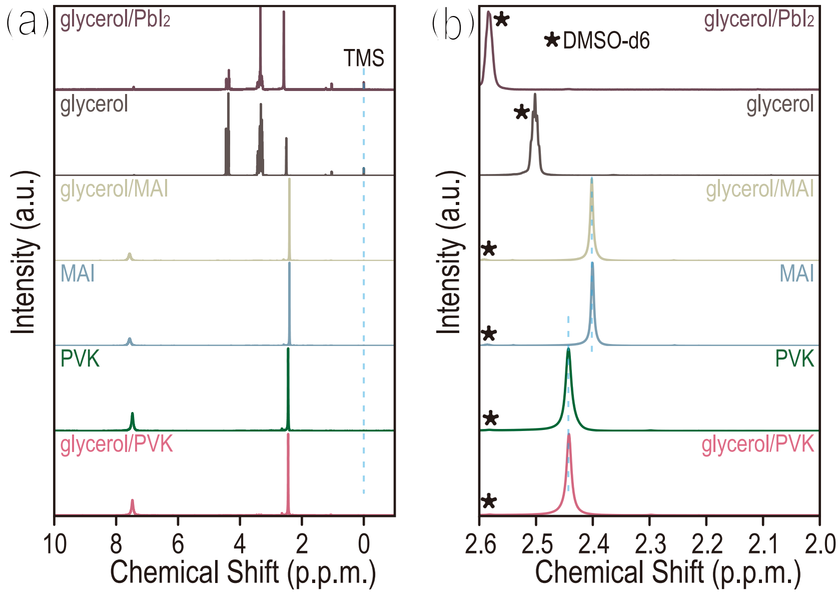

Figure S8. Liquid-state ^1H magnetic resonance (^1H-NMR) spectra of samples dissolved in dimethyl sulfoxide (DMSO-d6) with (a) survey (-1 - 10 ppm) and (b) magnification (2.0 - 2.6 ppm) of scale.

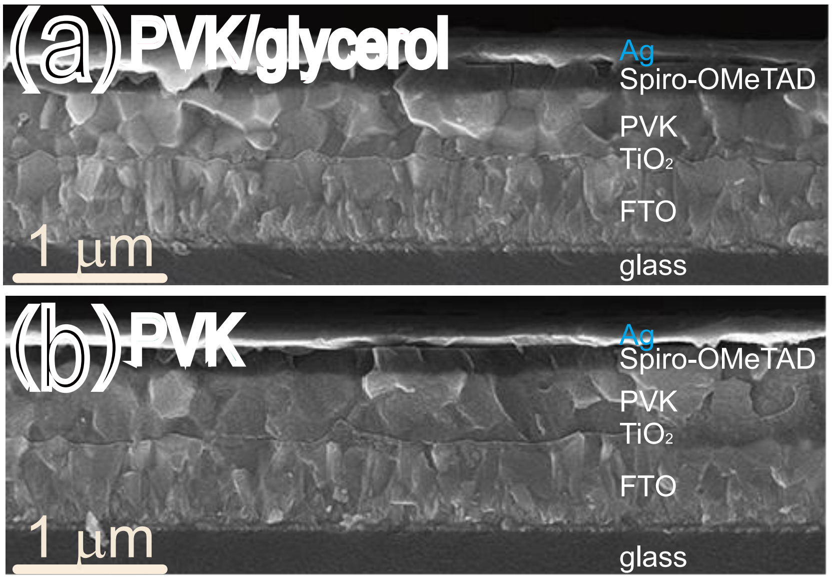

Figure S9. Cross sectional SEM images of PSCs with structure of $FTO/TiO_2/MAPbI_3$/Spiro-OMeTAD/Ag in which $MAPbI_3$ is doped (a) with and (b) without glycerol (0.15 mg/mL).

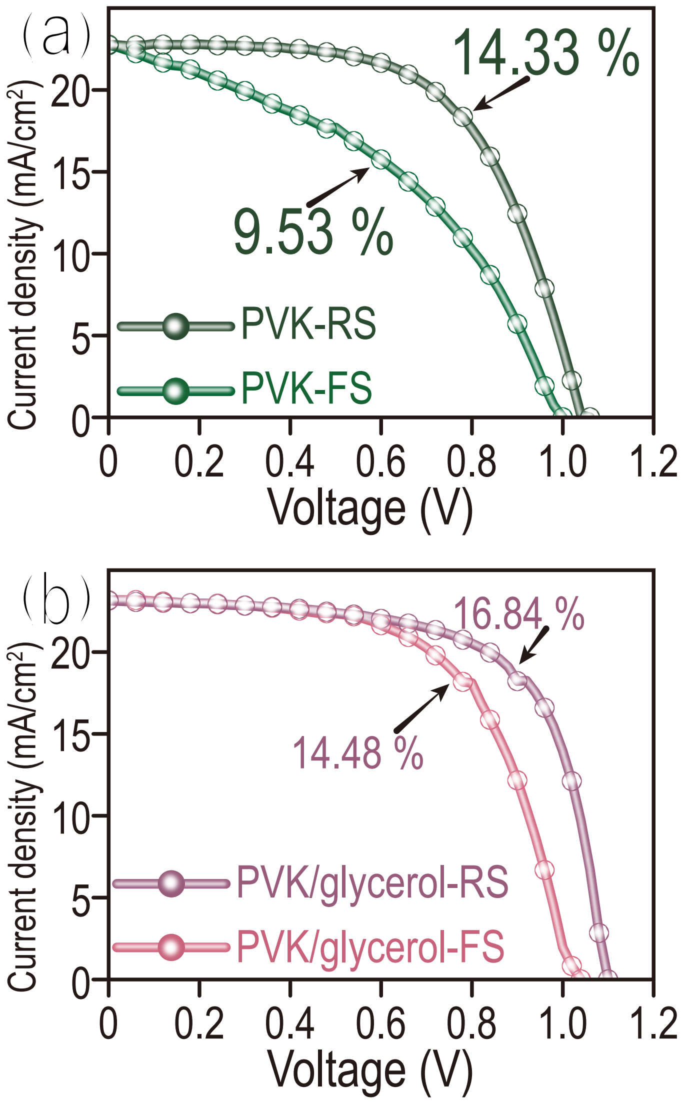

Figure S10. Hysteresis effects of (a) pristine and (b) glycerol doped PSCs.

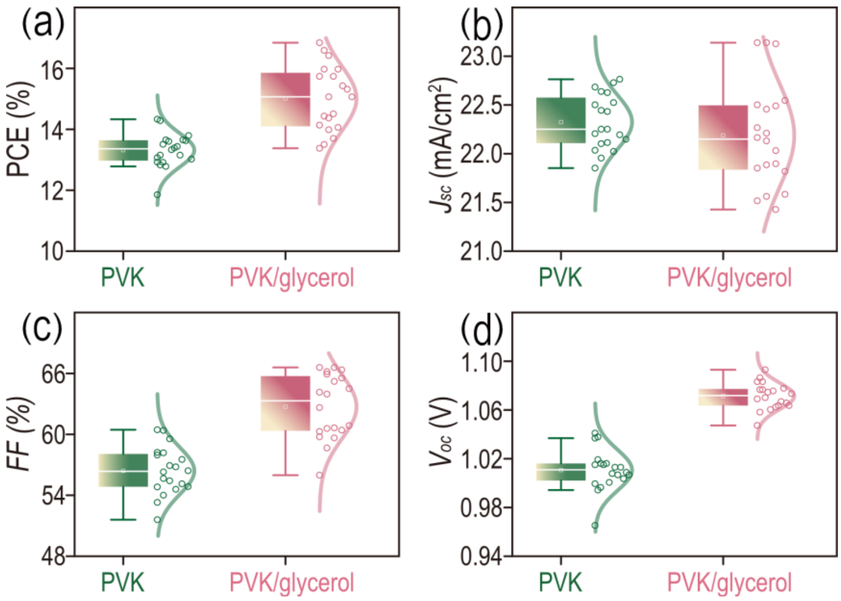

Figure S11. Statistical results of *J-V* parameters including (a) PCE, (b) J_{sc}, (c) *FF* and (d) V_{oc} of twenty PSCs doped with and without glycerol.

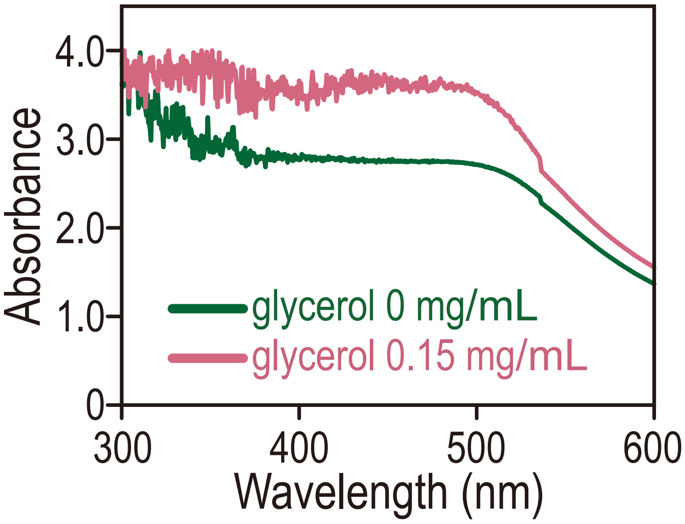

Figure S12. UV-vis spectra of $MAPbI_3$ film with and without glycerol in range from 300 to 600 nm.

Table S1. The parameters of perovskite films with and without glycerol extracted from the XPS spectra and Tauc plot.

	$E_{cut\text{-}off}$ [eV]	WF [eV]	E_{edge} [eV]	E_{CB} [eV]

Glycerol-doped	17.70	4.53	1.01	3.96
Pristine	17.64	4.52	0.94	3.89

Table S2. The photovoltaic parameters for glycerol-doped (0.15 mg/mL) and pristine devices.

	V_{OC} [V]	J_{SC} [mA /cm^2]	FF [%]	PCE [%]
Glycerol-doped	1.093	23.14	66.59	16.84
Pristine	1.041	22.76	60.45	14.33

Table S3. Fitted R_s and R_{rec} data of EIS curves of PSCs incorporated with and without glycerol.

	PSCs	PSCs + glycerol
R_s / Ω	29.99	19.33
R_{rec} / kΩ	269.89	582.36
